# *LIN28A* gene polymorphisms confer Wilms tumour susceptibility: A four‐centre case‐control study

**DOI:** 10.1111/jcmm.14561

**Published:** 2019-07-23

**Authors:** Zhenjian Zhuo, Wen Fu, Jiabin Liu, Jiwen Cheng, Haixia Zhou, Jiao Zhang, Jin‐Hong Zhu, Huimin Xia, Guochang Liu, Jing He

**Affiliations:** ^1^ Department of Pediatric Surgery, Guangzhou Institute of Pediatrics, Guangdong Provincial Key Laboratory of Research in Structural Birth Defect Disease, Guangzhou Women and Children's Medical Center Guangzhou Medical University Guangzhou China; ^2^ School of Chinese Medicine, Faculty of Medicine The Chinese University of Hong Kong Hong Kong China; ^3^ Department of Pediatric Surgery The Second Affiliated Hospital of Xi'an Jiaotong University Xi'an China; ^4^ Department of Hematology The Second Affiliated Hospital and Yuying Children's Hospital of Wenzhou Medical University Wenzhou China; ^5^ Department of Pediatric Surgery The First Affiliated Hospital of Zhengzhou University Zhengzhou China; ^6^ Department of Clinical Laboratory Molecular Epidemiology Laboratory Harbin Medical University Cancer Hospital Harbin China

**Keywords:** case‐control study, *LIN28A*, polymorphism, susceptibility, Wilms tumour

## Abstract

Wilms tumour is a renal malignancy that commonly occurs in children. *LIN28A* gene overexpression has been reported to be involved in various human malignancies, while its roles in Wilms tumour risk are still under investigation. Here, we genotyped four *LIN28A* polymorphisms in 355 Wilms tumour patients and 1070 healthy controls from four hospitals in China. The genotyped single nucleotide polymorphisms (SNPs) include the following: rs3811464 G>A, rs3811463 T>C, rs34787247 G>A and rs11247957 G>A. Overall, we found that rs3811463 T>C and rs34787247 G>A were associated with increased risk of Wilms tumour. Combination analysis of risk genotypes showed that, compared to non‐carriers, subjects with 1 risk genotype and 1‐3 risk genotypes were more likely to develop Wilms tumour, with an adjusted odds ratio (OR) of 1.58 and 1.56, respectively. Stratified analysis further demonstrated that the risk effect remained prominent in some subgroups. We also found that presence of 1‐3 risk genotypes was associated with Wilms tumour risk in subgroups > 18 months of age, females, males and those with clinical stage I + II diseases. Furthermore, expression quantitative trait locus (eQTL) analysis indicated that rs3811463 C allele was significantly associated with increased transcripts of *LIN28A* gene. These findings suggest that *LIN28A* gene polymorphisms may be associated with increased predisposition to Wilms tumour.

## 1 INTRODUCTION

Wilms tumour is a common paediatric renal cancer worldwide, which mostly affects children.^1^ The prevalence of Wilms tumour is nearly 1 out of 10 000 in North America.^2^, ^3^ Despite substantial advances in the managements of Wilms tumour, prognosis of relapsed patients remains poor even after re‐treated with chemotherapy and surgery alone; a 4‐year overall survival rate is less than 50% for this subgroup of patients.^4^ To be note, previous study has been shown that *VHL*, *PBRM1*, *SETD2* and *BAP1* are most frequently mutated in regular kidney cancers.^5^ However, because of the different genome mutation landscape from the adult counterparts, there is a critical need to identify more causal genetic variations in Wilms tumour.

Lin28 is an RNA‐binding protein. It is implicated in cell growth, glucose metabolism and pluripotency, through regulating the biogenesis of miRNAs. In mammals, *LIN28* gene encodes two RNA‐binding paralogs, Lin28A and Lin28B. Lin28A/B inhibits let‐7 microRNAs maturation and then promotes translation of certain target mRNAs of let‐7.^6^ The key mechanism by which Lin28A represses let‐7 biogenesis is that cytoplasmic Lin28A targets precursor form of let‐7 (pre‐let‐7) and then recruits TUTase 4 to induce oligo‐uridylation of pre‐let‐7. Polyuridylation then facilitates pre‐let‐7 destabilization and ultimately decreases the level of mature let‐7.^7^

The molecular mechanism underlying the Lin28A‐mediated carcinogenesis has been elucidated, to some extent. However, the contribution of *LIN28A* gene single nucleotide polymorphisms (SNPs) to Wilms tumour risk has not been investigated previously. Using clinical samples from a large multi‐centre study, we attempted to determine the associations between *LIN28A* SNPs and Wilms tumour risk. We identified rs3811463 T>C and rs34787247 G>A polymorphisms in *LIN28A* significantly associated with Wilms tumour risk. These data implicate polymorphisms in *LIN28A* gene as an important mechanism of Wilms tumour tumorigenesis.

## 2 MATERIALS AND METHODS

### 2.1 Study subjects

Participants in the study were recruited from four hospitals in China: Guangzhou Women and Children's Medical Center,^8^ The First Affiliated Hospital of Zhengzhou University, The Second Affiliated Hospital and Yuying Children's Hospital of Wenzhou Medical University, and The Second Affiliated Hospital of Xi'an Jiao Tong University. Patients were newly diagnosed and histologically confirmed with Wilms tumour. Eligible controls were chosen from the same residing areas as cases and were frequency‐matched to cases on age, sex in each participating centre. In all, 355 cases and 1070 controls 9 were recruited. All participants provided written informed consents. Demographic information was collected by trained interviewers. Detailed information on participant selection was reported in our previous publications. This study was approved by the institutional review boards of all participating hospitals.

### 2.2 Polymorphism selection and genotyping

A total of four *LIN28A* SNPs with potential function were included in the analysis, following standard selection criteria. The locations of these SNPs in the *LIN28A* are as below: rs3811464 G>A in the upstream, rs3811463 T>C, rs34787247 G>A and rs11247957 G>A are all in the 3’‐UTR. The details of these four SNPs are shown in Table S1 in Appendix S1. DNA was mainly extracted from study participants using a TIANamp Blood DNA Kit (TianGen Biotech Co Ltd). Genotyping was carried out following the manufacturer's instructions by TaqMan methodology. We used water as negative controls to ensure genotyping accuracy. 10% random selected samples were re‐genotyped with 100% concordance rates.

### 2.3 Statistical analysis

The chi‐square test was firstly applied to assess each SNP for deviation from the Hardy‐Weinberg equilibrium (HWE) among the controls. The differences in the distribution of categorical variables between the cases and controls were analysed using the two‐sided chi‐square test. We evaluated the association between the four selected SNPs and Wilms tumour risk by using multivariable logistic regression analysis. The association results are presented as odds ratios (ORs) with 95% confidence intervals (CIs), with adjustment for gender and age. We calculated the false‐positive report probability (FPRP) to test for false‐positive associations. A probability of <.2 was considered a noteworthy association. We also conducted the expression quantitative trait loci (eQTL) analysis using GTEx portal website (http://www.gtexportal.org/home/), to predict the influence of SNPs on expression level of *LIN28A*.^10^ Statistical significance was defined when *P* < .05. The SAS release 9.1 (SAS Institute) was adopted to conduct all statistical analyses.

## 3 RESULTS

### 3.1 Population description

Table S2 in Appendix S1 gives information on the baseline characteristics of the included cases and controls. Similar distributions of age (*P* = .131) and gender (*P* = .182) were observed between cases and controls. As to clinical stages of cases, 119 (33.52%), 92 (25.92%), 79 (22.25%) and 47 (13.24%) patients were classified into clinical stage I, II, III and IV, respectively, according to the NWTS‐5 criteria.^11^ Clinical stage was unable to determine for a small subset of 18 (5.07%) patients.

### 3.2 Risks associated with the *LIN28A* polymorphisms

The associations of all the four *LIN28A* SNPs (rs3811464 G>A, rs3811463 T>C, rs34787247 G>A and rs11247957 G>A) with Wilms tumour risk were listed in Table 1. In controls, all the four SNPs were consistent with HWE (HWE *P* > .05). Our results indicated that rs3811463 CC/TC genotype showed significant association with the risk of Wilms tumour. Individuals with this genotype had a 1.33‐fold increased risk of Wilms tumour (95% CI = 1.03‐1.73, *P* = .031) when compared with those with TT genotype. We also identified that rs34787247 A allele could confer to increased risk of Wilms tumour (AA vs GG: adjusted OR = 2.41, 95% CI = 1.22‐4.79; AA vs GG/GA: adjusted OR = 2.31, 95% CI = 1.17‐4.57). The individual rs3811464 G>A and rs11247957 G>A variant was not associated with altered risk of Wilms tumour. The combined effect of risk genotypes on Wilms tumour risk was then evaluated. Compared to individuals without risk genotype, those with 1 risk genotype had a 1.58‐fold increased risk of Wilms tumour (95% CI = 1.23‐2.03, *P* = .0004), and those carrying 1‐3 of these genotypes had a 1.56‐fold increased risk (95% CI = 1.22‐1.99, *P* = .0003).

**Table 1:** Association of *LIN28A* polymorphisms with Wilms tumour susceptibility

Genotype	Cases (N = 355)	Controls (N = 1070)	*P* a	Crude OR (95% CI)	*P*	Adjusted OR (95% CI) b	*P* b
rs3811464 G>A (HWE = 0.063)							
GG	261 (73.52)	790 (73.83)		1.00		1.00	
GA	81 (22.82)	250 (23.36)		0.98 (0.74‐1.31)	.894	0.98 (0.73‐1.30)	.872
AA	13 (3.66)	30 (2.80)		1.31 (0.67‐2.55)	.424	1.33 (0.68‐2.58)	.408
Additive			.709	1.04 (0.83‐1.32)	.712	1.04 (0.83‐1.31)	.716
Dominant	94 (26.48)	280 (26.17)	.908	1.02 (0.77‐1.33)	.908	1.01 (0.77‐1.33)	.922
Recessive	342 (96.34)	1040 (97.20)	.413	1.32 (0.68‐2.56)	.414	1.33 (0.69‐2.59)	.396
rs3811463 T>C (HWE = 0.530)							
TT	240 (67.61)	785 (73.36)		1.00		1.00	
TC	103 (29.01)	260 (24.30)		1.30 (0.99‐1.70)	.060	1.31 (1.00‐1.71)	.053
CC	12 (3.38)	25 (2.34)		1.57 (0.78‐3.17)	.209	1.60 (0.79‐3.24)	.190
Additive			.031	**1.28 (1.02‐1.60)**	**.031**	**1.29 (1.03‐1.62)**	**.026**
Dominant	115 (32.39)	285 (26.64)	.036	**1.32 (1.02‐1.71)**	**.037**	**1.33 (1.03‐1.73)**	**.031**
Recessive	343 (96.62)	1045 (97.66)	.284	1.46 (0.73‐2.94)	.286	1.49 (0.74‐3.00)	.266
rs34787247 G>A (HWE = 0.390)							
GG	255 (71.83)	821 (76.73)		1.00		1.00	
GA	85 (23.94)	229 (21.40)		1.20 (0.90‐1.59)	.222	1.20 (0.90‐1.59)	.220
AA	15 (4.23)	20 (1.87)		**2.42 (1.22‐4.79)**	**.012**	**2.41 (1.22‐4.79)**	**.012**
Additive			.022	**1.32 (1.05‐1.67)**	**.017**	**1.33 (1.05‐1.67)**	**.017**
Dominant	100 (28.17)	249 (23.27)	.063	1.29 (0.99‐1.70)	.063	1.29 (0.99‐1.70)	.063
Recessive	340 (95.77)	1050 (98.13)	.013	**2.32 (1.17‐4.57)**	**.016**	**2.31 (1.17‐4.57)**	**.016**
rs11247957 G>A (HWE = 0.554)							
GG	341 (96.06)	1032 (96.45)		1.00		1.00	
GA	13 (3.66)	38 (3.55)		1.04 (0.55‐1.97)	.916	1.05 (0.55‐2.00)	.882
AA	1 (0.28)	0 (0.00)		/	/	/	/
Additive			.220	1.19 (0.65‐2.16)	.569	1.20 (0.66‐2.18)	.551
Dominant	14 (3.94)	38 (3.55)	.733	1.12 (0.60‐2.08)	.733	1.13 (0.60‐2.11)	.706
Recessive	354 (99.72)	1070 (100.00)	.082	/	/	/	/
Risk genotypes c							
0	148 (41.69)	562 (52.52)	.005	1.00		1.00	
1	175 (49.30)	424 (39.63)		**1.57 (1.22‐2.02)**	**.0005**	**1.58 (1.23‐2.03)**	**.0004**
2	29 (8.17)	74 (6.92)		1.49 (0.93‐2.37)	.095	1.52 (0.95‐2.42)	.081
3	3 (0.85)	10 (0.93)		1.14 (0.31‐4.19)	.845	1.13 (0.31‐4.16)	.856
0	148 (41.69)	562 (52.52)		1.00		1.00	
1‐3	207 (58.31)	508 (47.48)	.0004	**1.55 (1.21‐1.97)**	**.0004**	**1.56 (1.22‐1.99)**	**.0003**

a χ^2^ test for genotype distributions between Wilms tumour patients and controls.

b Adjusted for age and gender.

c Risk genotypes were rs3811464 AA, rs3811463 TC/CC, rs34787247 GA/AA and rs11247957 GA/AA.

The results were in bold if the 95% CI excluded 1 or P<0.05.

### 3.3 Stratification analysis

Table 2 presents the association results of *LIN28A* gene polymorphisms with Wilms tumour risk in a particular subgroup, stratified by age, gender and clinical stages. We found that the increased risk associated with the rs3811463 TC/CC variant genotype was more pronounced in children with age ≤ 18 months (adjusted OR = 1.70, 95% CI = 1.11‐2.60), as well as patients in clinical stages I + II (adjusted OR = 1.59, 95% CI = 1.16‐2.18). Compared to the rs34787247 GG genotype, the risk effect of GA/AA genotypes was more predominant for children > 18 months of age (adjusted OR = 1.71, 95% CI = 1.22‐2.38, *P* = .002). After combining the risk genotypes, we observed that the present of 1‐3 risk genotypes were more likely to associated with tumour risk in subgroups of age > 18 month (adjusted OR = 1.70, 95% CI = 1.25‐2.32, *P* = .0007), females (adjusted OR = 1.48, 95% CI = 1.03‐2.11, *P* = .035), males (adjusted OR = 1.64, 95% CI = 1.18‐2.28, *P* = .004) and clinical stage I + II (adjusted OR = 1.72, 95% CI = 1.27‐2.33, *P* = .0005).

**Table 2:** Stratification analysis of protective genotypes and Wilms tumour susceptibility

Variables	rs3811463 (cases/controls)		AOR (95% CI)^a^	*P* a	rs34787247 (cases/controls)		AOR (95% CI)^a^	*P* a	Combined genotypes (cases/controls)		AOR (95% CI)^a^	*P* a
	TT	TC/CC			GG	GA/AA			0	1‐3		
Age, month												
≤18	79/317	46/108	**1.70 (1.11‐2.60)**	**.014**	100/319	25/106	0.76 (0.46‐1.24)	.264	56/222	69/203	1.34 (0.90‐2.00)	.154
>18	161/468	69/177	1.16 (0.83‐1.62)	.377	155/502	75/143	**1.71 (1.22‐2.38)**	**.002**	92/340	138/305	**1.70 (1.25‐2.32)**	**.0007**
Gender												
Females	113/341	50/107	1.41 (0.95‐2.10)	.090	119/340	44/108	1.16 (0.77‐1.75)	.469	73/244	90/204	**1.48 (1.03‐2.11)**	**.035**
Males	127/444	65/178	1.27 (0.90‐1.80)	.170	136/481	56/141	1.42 (0.99‐2.05)	.059	75/318	117/304	**1.64 (1.18‐2.28)**	**.004**
Clinical stages												
I + II	134/785	77/285	**1.59 (1.16‐2.18)**	**.004**	152/821	59/249	1.28 (0.92‐1.79)	.144	83/562	128/508	**1.72 (1.27‐2.33)**	**.0005**
III + IV	97/785	29/285	0.83 (0.53‐1.28)	.398	88/821	38/249	1.42 (0.95‐2.14)	.090	60/562	66/508	1.22 (0.84‐1.77)	.289

a Adjusted for age and gender, omitting the corresponding stratification factor. The results were in bold if the 95% CI excluded 1 or *P* < 0.05.

### 3.4 False‐positive report probability results

We further calculated false‐positive report probability (FPRP) values for the significant findings, with results shown in Table S3 in Appendix S1. At the prior probability level of .1, significance of the statistically significant findings for rs3811463 T>C (TC/CC vs GG) and rs34787247 G>A disappears. As to the stratification analyses, association with rs3811463 T>C in clinical stages I + II patients and association with rs34787247 G>A in subjects older than 18 months old remain noteworthy. As for combined risk genotype analysis, we found that significant findings for the 1 vs 0 and 1‐3 vs 0 genotypes remained noteworthy. FPRP analysis for stratification analyses of 1‐3 vs 0 polymorphism also revealed a noteworthy result in subgroup of age > 18, males and stages I + II.

### 3.5 Expression quantitative trait locus (eQTL) analysis

To further assess the putative functional relevance of *LIN28A* rs3811463 T>C, we evaluated the correlation between rs3811463 T>C polymorphism and *LIN28A* expression in transformed fibroblasts tissues. We found that the risk allele (C) of rs3811463 could enhance *LIN28A* expression significantly (Figure S1 in Appendix S1).

## 4 DISCUSSION

Currently, there still exists a substantial knowledge gap between SNPs and Wilms tumour susceptibility. Thus, identifying more polymorphisms helps to further define the full spectrum of genetic variations that contributes to Wilms tumour susceptibility. Here, we evaluated the association between the *LIN28A* polymorphisms and Wilms tumour susceptibility in Chinese patients.

*LIN28A* gene is located to chromosome 1p36.11. Until recently, several studies regarding polymorphisms in *LIN28A* gene and cancer risk have been published. Permuth‐Wey et al found that rs11247946 and rs12728900 of *LIN28A* predispose to epithelial ovarian cancer susceptibility in European ancestry.^12^ In a study conducted in China, Zhang et al explored association between six genetic variants in let‐7/Lin28 and oral cavity cancer risk with 384 cases and 731 controls, and they detected a protective effect of *LIN28B* rs221636 on oral cavity cancer. However, they failed to observe the association with the risk of oral cavity cancer for *LIN28A* rs4659441 and rs3811463.^13^ Sung et al conducted a two genome‐wide association study in East Asian with 5066 breast cancer cases and 4337 controls involving Koreans and Chinese. By investigating 237 SNPs in 32 genes involved in microRNA biogenesis‐related pathways, the authors found that none of studied SNPs were associated with breast cancer risk, including 7 SNPs in the *LIN28A* (rs11247954, rs6683792, rs4274112, rs6598964, rs12728900, rs4659441, rs3811463).^14^ The different impacts of *LIN28A* polymorphisms on cancers indicated that the role of *LIN28A* polymorphisms in cancer risk may depend on cancer types, ethnicities and study sample sizes. Therefore, identifying the role of *LIN28A* SNPs on certain cancer and certain population is indispensible.

Our previous epidemiological study showed that the *LIN28B* polymorphisms may be able to modify Wilms tumour susceptibility in Chinese children.^15^ As *LIN28A* is quite similar to *LIN28B* in either structure or cellular function, it is biologically plausible that *LIN28A* polymorphisms may also predispose to Wilms tumour. Our results indicated that rs3811463 CC/TC genotype and rs34787247 A allele confer an increased risk of Wilms tumour. We also detected significant increased risks of Wilms tumour in subjects carrying 1 risk genotype and 1‐3 risk genotypes, compared to individuals without risk genotype. However, we failed to observe significant association for rs3811464 G>A and rs11247957 G>A variant. Moreover, FPRP analysis indicated that some significant findings no longer remain noteworthy at the prior probability level of 0.1. These results indicate that some findings might be significant just by chance because of relative small sample size. Findings from eQTL analysis showed that rs3811463 C allele is associated with up‐regulated *LIN28A* expression, thus might potentially contribute to increased Wilms tumour risk.

This study leads a vital part as a pioneer in exploring the association between *LIN28A* gene SNPs and Wilms tumour risk. Merit of this study is its origination on the first large‐scale multi‐centre‐based analysis. The study also accompanies some minor limitations. First, even though our sample size is considerable given the low incidence of Wilms tumour, the sample size is still not enough to provide a strong statistical power. Some negative results obtained here might need to be further validated, especially for stratification analysis. Second, only four SNPs were analysed in the current study. There might be more SNPs in *LIN28A* gene associated with Wilms tumour risk. Third, all the included subjects were Chinese Han population although selected from four different hospitals. The findings might not be applicable to other populations. Last, we only took into consideration of the genetic factors because the information of environmental factors was not accessible. Thus, we are unable to determine whether the analysed *LIN28A* polymorphism is modified by certain environmental factors.

In all, we performed a multi‐centre study to investigate the association between *LIN28A* polymorphisms and Wilms tumour risk in Chinese population. Our findings for the first time provided the insight into the potential role of *LIN28A* gene polymorphisms in Wilms tumour risk. Nevertheless, our conclusion based on genetic analysis is far from enough to fully elucidate the aetiology of Wilms tumour. Comprehensive analysis, considering both genetic factors, environmental factors and genetic‐environmental interactions, will help to reveal the pathogenesis of Wilms tumour.

## CONFLICT OF INTEREST

The authors declare no competing financial interests.

## AUTHOR'S CONTRIBUTION

Z. Zhuo, W. Fu, J. Liu, G. Liu and J. He designed and performed the study and wrote the manuscript; W. Fu, J. Cheng, H. Zhou, J. Zhang and H. Xia collected the samples and information; Z. Zhuo, J. Zhu and J. He participated in analysing data; and W. Fu, G. Liu and J. He co‐ordinated the study over the entire time. All authors reviewed the final manuscript.

## Data Availability

All the data were available upon request.
