# Supplementary material for: LIN28A gene polymorphisms confer Wilms tumour susceptibility: A four‐centre case‐control study
Source: J Cell Mol Med. 2019 Jul 23;23(10):7105–10. doi: 10.1111/jcmm.14561 (PMC6787499; doi:10.1111/jcmm.14561)
Supplement: Supplementary file 1 [file JCMM-23-7105-s001.doc]

| **Table S1**. SNPs captured by the four selected *LIN28A* potentially functional SNPs as predicted by SNPinfo software | | | | | | | | | | | |
| --- | --- | --- | --- | --- | --- | --- | --- | --- | --- | --- | --- |
| **rs** | **Chr.** | **Allele** | **LDsnp** | **Pop/LD** | **TFBS** | **Splicing**  **(ESE or ESS)** | **miRNA**  **(miRanda)** | **Nearby Gene** | **Allele** | **Asian** | **CHB** |
| **rs11247957 ^a^** | **1** | **A/G** | **rs11247957** | **1** | **Y** | **Y** | **Y** | **LIN28** | **G** | **--** | **0.981** |
| **rs34787247 ^a^** | **1** | **A/G** | **rs34787247** | **1** | **Y** | **--** | **Y** | **LIN28** | **G** | **--** | **0.864** |
| rs11247955 | 1 | A/G | rs3811463 | CHB/0.916 | -- | -- | -- | LIN28 | G | 0.894 | 0.856 |
| rs12741800 | 1 | C/T | rs3811463 | CHB/1 | -- | -- | Y | HMGN2 | T | 0.877 | 0.807 |
| rs17261915 | 1 | C/T | rs3811463 | CHB/0.916 | Y | -- | -- | LIN28||DHDDS | T | 0.886 | 0.821 |
| rs2219320 | 1 | C/T | rs3811463 | CHB/0.916 | -- | -- | -- | HMGN2||LOC100128503 | T | 0.885 | 0.856 |
| rs35015532 | 1 | A/G | rs3811463 | CHB/0.916 | -- | -- | -- | LIN28 | A | -- | 0.827 |
| **rs3811463** | **1** | **T/C** | **rs3811463** | **1** | **Y** | **--** | **Y** | **LIN28** | **T** | **0.871** | **0.844** |
| rs11247950 | 1 | G/T | rs3811464 | CHB/1 | -- | -- | -- | ZNF683||LIN28 | G | 0.783 | 0.815 |
| rs11591111 | 1 | A/C | rs3811464 | CHB/1 | -- | -- | -- | ZNF683||LIN28 | C | 0.800 | 0.815 |
| rs12122703 | 1 | A/G | rs3811464 | CHB/1 | Y | -- | -- | ZNF683||LIN28 | A | -- | 0.815 |
| rs12722898 | 1 | A/G | rs3811464 | CHB/0.922 | -- | -- | -- | ZNF683||LIN28 | G | 0.800 | 0.815 |
| rs12724751 | 1 | A/T | rs3811464 | CHB/0.931 | -- | -- | -- | ZNF683||LIN28 | T | -- | 0.807 |
| rs12747426 | 1 | C/G | rs3811464 | CHB/1 | Y | -- | -- | ZNF683||LIN28 | C | 0.820 | 0.800 |
| rs17163904 | 1 | C/T | rs3811464 | CHB/0.846 | -- | -- | -- | LIN28 | T | 0.827 | 0.839 |
| **rs3811464** | **1** | **G/A** | **rs3811464** | **1** | **Y** | **--** | **--** | **ZNF683||LIN28** | **G** | **--** | **0.815** |
| rs4360511 | 1 | A/G | rs3811464 | CHB/1 | -- | -- | -- | ZNF683||LIN28 | G | 0.800 | 0.815 |
| rs6683792 | 1 | C/T | rs3811464 | CHB/0.93 | -- | -- | -- | LIN28 | C | 0.829 | 0.802 |
| rs7532866 | 1 | A/G | rs3811464 | CHB/0.804 | -- | -- | -- | LIN28 | A | 0.829 | 0.829 |
| rs7552060 | 1 | A/G | rs3811464 | CHB/0.961 | -- | -- | -- | ZNF683||LIN28 | G | 0.800 | 0.802 |
| rs7556500 | 1 | A/T | rs3811464 | CHB/1 | -- | -- | -- | ZNF683||LIN28 | A | 0.800 | 0.815 |
| SNP, single nucleotide polymorphism; LD, linkage disequilibrium; TFBS, transcription factor binding sites; ESE, exonic splicing enhancer; ESS, exonic splicing silencer; CHB, Han Chinese in Beijing, China.  ^a^ Using data from 1000 Genomes (https://www.ncbi.nlm.nih.gov/variation/tools/1000genomes/) for CHB population. | | | | | | | | | | | |

| **Table S2**. Frequency distribution of selected variables in Wilms tumor patients and controls | | | | | |
| --- | --- | --- | --- | --- | --- |
| Variables | Cases (n=355) | | Controls (n=1070) | | *P^a^* |
|  | No. | % | No. | % |  |
| Age range, month | 1-148.63 | | 0.03-156 | | 0.131 |
| Mean ± SD | 30.67 ± 23.96 | | 32.27 ± 26.89 | |  |
| ≤18 | 125 | 35.21 | 425 | 39.72 |  |
| >18 | 230 | 64.79 | 645 | 60.28 |  |
| Gender |  |  |  |  | 0.182 |
| Female | 163 | 45.92 | 448 | 41.87 |  |
| Male | 192 | 54.08 | 622 | 58.13 |  |
| Clinical stages |  |  |  |  |  |
| I | 119 | 33.52 |  |  |  |
| II | 92 | 25.92 |  |  |  |
| III | 79 | 22.25 |  |  |  |
| IV | 47 | 13.24 |  |  |  |
| NA | 18 | 5.07 |  |  |  |
| ^a^ Two-sided χ^2^ test for distributions between Wilms tumor patients and cancer-free controls. | | | | | |

| **Table S3**. False-positive report probability analysis for the significant associations between *LIN28A* genotypes and Wilms tumor susceptibility | | | | | | | | |
| --- | --- | --- | --- | --- | --- | --- | --- | --- |
| Genotype | Crude OR  (95% CI) | *P* ^a^ | Statistical power ^b^ | Prior probability | | | | |
|  |  |  |  | 0.25 | 0.1 | 0.01 | 0.001 | 0.0001 |
| rs3811463 T>C | | | | | | | | |
| TC/CC vs. GG | 1.32 (1.02-1.71) | 0.037 | 0.838 | **0.116** | 0.283 | 0.813 | 0.978 | 0.998 |
| ≤18 | 1.71 (1.12-2.61) | 0.013 | 0.275 | **0.126** | 0.302 | 0.826 | 0.980 | 0.998 |
| Stage I+II | 1.58 (1.16-2.16) | 0.004 | 0.371 | **0.030** | **0.084** | 0.504 | 0.911 | 0.990 |
| rs34787247 G>A | | | | | | | | |
| AA vs. GG | 2.42 (1.22-4.79) | 0.012 | 0.088 | 0.282 | 0.541 | 0.928 | 0.992 | 0.999 |
| AA vs. GG/GA | 2.32 (1.17-4.57) | 0.016 | 0.105 | 0.309 | 0.573 | 0.937 | 0.993 | 0.999 |
| AA/AG vs. GG | | | | | | | | |
| >18 | 1.70 (1.22-2.37) | 0.002 | 0.233 | **0.023** | **0.065** | 0.433 | 0.885 | 0.987 |
| Risk genotypes | | | | | | | | |
| 1 vs. 0 | 1.57 (1.22-2.02) | 0.0005 | 0.429 | **0.003** | **0.010** | **0.103** | 0.538 | 0.921 |
| 1-3 vs. 0 | 1.55 (1.21-1.97) | 0.0004 | 0.400 | **0.003** | **0.009** | **0.090** | 0.499 | 0.909 |
| >18 | 1.67 (1.23-2.27) | 0.001 | 0.251 | **0.012** | **0.035** | 0.283 | 0.799 | 0.976 |
| Females | 1.48 (1.03-2.11) | 0.035 | 0.539 | **0.162** | 0.367 | 0.864 | 0.985 | 0.998 |
| Males | 1.64 (1.18-2.28) | 0.004 | 0.311 | **0.033** | **0.092** | 0.527 | 0.918 | 0.991 |
| Stage I+II | 1.71 (1.26-2.31) | 0.0005 | 0.208 | **0.007** | **0.021** | **0.192** | 0.706 | 0.960 |
| ^a^ χ^2^ test was used to calculate the genotype frequency distributions.  ^b^ Statistical power was calculated using the number of observations in the subgroup and the OR and *P* values in this table. | | | | | | | | |

**Figure S1**. Expression quantitative loci (eQTL) analysis of *LIN28A* gene rs3811463 T>C. The genotype of rs3811463 T>C and expression of *LIN28A*
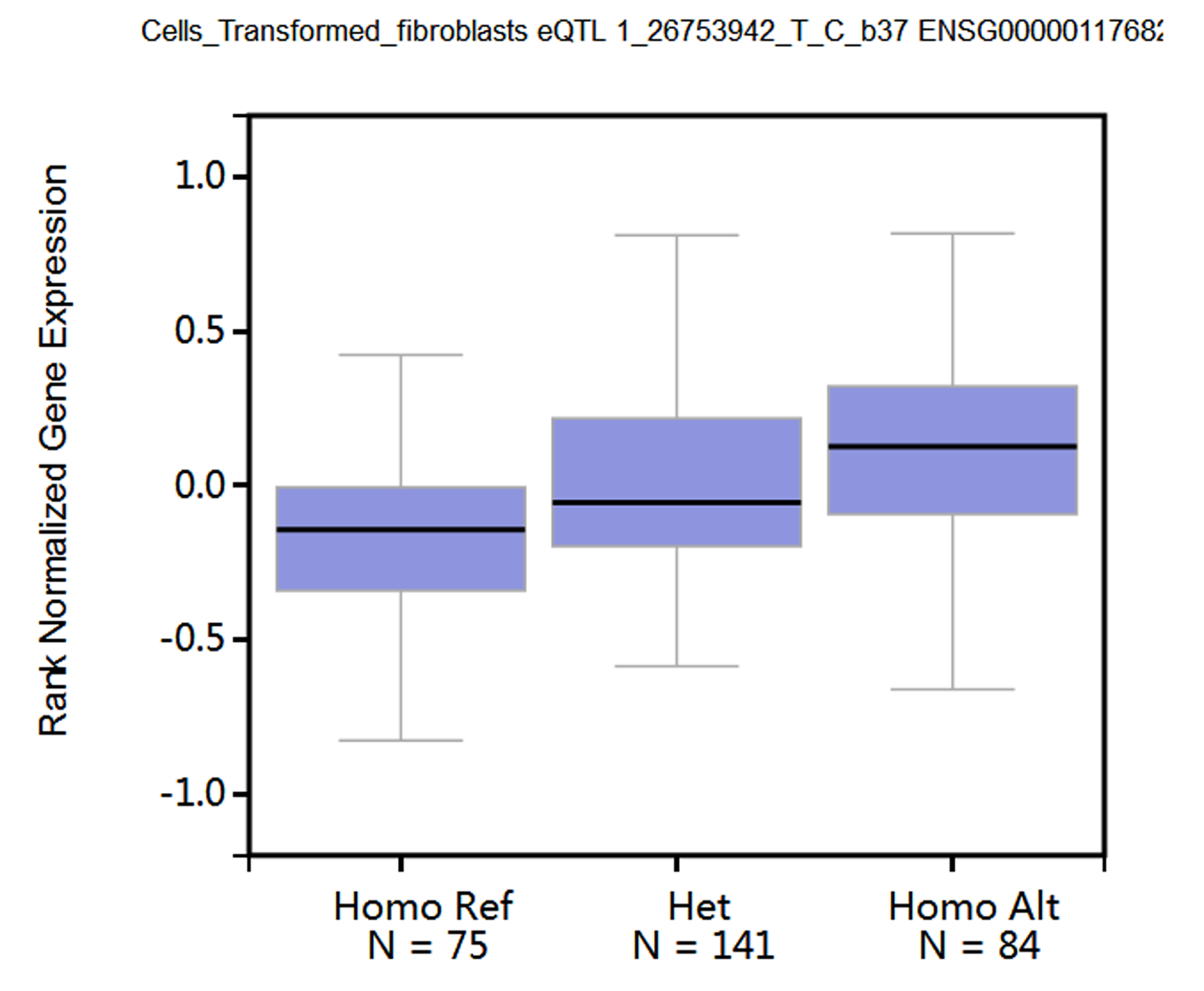
 gene in transformed fibroblasts tissues were searched based on the public database GTEx Portal (http://www.gtexportal.org/home/).
